# 17-β Estradiol up-regulates energy metabolic pathways, cellular proliferation and tumor invasiveness in ER+ breast cancer spheroids

**DOI:** 10.3389/fonc.2022.1018137

**Published:** 2022-11-07

**Authors:** Silvia Cecilia Pacheco-Velázquez, Ingrid Itzayanna Ortega-Mejía, Jorge Luis Vargas-Navarro, Joaquín Alberto Padilla-Flores, Diana Xochiquetzal Robledo-Cadena, Gabriela Tapia-Martínez, Ignacio Peñalosa-Castro, José Luis Aguilar-Ponce, Juan Carlos Granados-Rivas, Rafael Moreno-Sánchez, Sara Rodríguez-Enríquez

**Affiliations:** ^1^ Departamento de Bioquímica, Instituto Nacional de Cardiología, Ciudad de México, Mexico; ^2^ Laboratorio de Control Metabólico, Carrera de Biología, Facultad de Estudios Superiores Iztacala, Universidad Nacional Autónoma de México, Los Reyes Ixtacala, Hab, Tlalnepantla, Mexico; ^3^ Hospital Médica Sur, Area de Oncología, Ciudad de México, Mexico; ^4^ Laboratorio de Control Metabólico, Carrera de Medicina, Facultad de Estudios Superiores Iztacala, Universidad Nacional Autónoma de México, Los Reyes Ixtacala, Hab, Tlalnepantla, Mexico

**Keywords:** 17-β estradiol, ER+ breast cancer, OxPhos, glycolysis, anti-mitochondrial therapy, metastasis

## Abstract

Several biological processes related to cancer malignancy are regulated by 17-β estradiol (E2) in ER+-breast cancer. To establish the role of E2 on the atypical cancer energy metabolism, a systematic study analyzing transcription factors, proteins, and fluxes associated with energy metabolism was undertaken in multicellular tumor spheroids (MCTS) from human ER+ MCF-7 breast cancer cells. At E2 physiological concentrations (10 and 100 nM for 24 h), both ERα and ERβ receptors, and their protein target pS2, increased by 0.6-3.5 times *vs*. non-treated MCTS, revealing an activated E2/ER axis. E2 also increased by 30-470% the content of several transcription factors associated to mitochondrial biogenesis and oxidative phosphorylation (OxPhos) (p53, PGC1-α) and glycolytic pathways (HIF1-α, c-MYC). Several OxPhos and glycolytic proteins (36-257%) as well as pathway fluxes (48-156%) significantly increased being OxPhos the principal ATP cellular supplier (>75%). As result of energy metabolism stimulation by E2, cancer cell migration and invasion processes and related proteins (SNAIL, FN, MM-9) contents augmented by 24-189% *vs.* non-treated MCTS. Celecoxib at 10 nM blocked OxPhos (60%) as well as MCTS growth, cell migration and invasiveness (>40%); whereas the glycolytic inhibitor iodoacetate (0.5 µM) and doxorubicin (70 nM) were innocuous. Our results show for the first time using a more physiological tridimensional cancer model, resembling the initial stages of solid tumors, that anti-mitochondrial therapy may be useful to deter hormone-dependent breast carcinomas.

## Introduction

The estrogen positive (ER+) breast cancer is one of the most frequently diagnosed human cancers, becoming the main cause of death in women of reproductive age worldwide (1). Malignancy of ER+ breast cancer is associated to estrogen receptors (ERs) overexpression (2). The ERs (ERα and Erβ) belong to a steroid/nuclear receptor superfamily activated by 17β-estradiol (E2) (3) and by other ligands (estriol, estrone) but with lower affinity (4). ER/ligand complex promotes a monomer-to-dimer self-transition and nuclear localization (3). In consequence, ERs bind to DNA transcriptional regulatory regions called estrogen response elements (EREs) located in their target genes, increasing gene expression in response to E2.

The ER/E2 complex triggers several intracellular events linked to the overexpression of proteins associated to ER+ breast cancer cell proliferation (cyclin D1, p53, BRCA-1) and angiogenesis (VEGF-R2) (5–7). Recently, the role of ER/E2 complex has also emerged as a lipid metabolism regulator. In breast MCF-7 and T47D cancer cells, ER/E2 increases the mRNA level (4-times) of proteins associated with monounsaturated fatty acids biosynthesis like the stearoyl-CoA desaturase (8, 9), indicating its role as anabolic inducer. E2 also augments the mRNA level (3-times) of OCTN2, a carnitine associated-carrier protein located in plasma membrane to supplying carnitine for mitochondrial β-oxidation (8, 9), which suggests a role for E2 as catabolic key primary regulator.

Scarce information is available regarding the role of E2 on cancer energy metabolism. In this regard, transcriptomic analysis reveals that E2 increases by 2-7 times the mRNA level of some glycolytic (HK-I and -II, PFK-2 and LDH-A) and mitochondrial (ND1 and COX-IV) enzymes in MCF-7 and T47D ER+-breast cancer cells (10, 11). E2 also increases the mRNA level of the mitochondrial biogenesis transcription factors PGC1-α and TFAM in breast MCF-7 and lung H1793 carcinoma cells (12). Unfortunately, transcriptomic results were not accompanied by experimental analysis of enzyme activities and fluxes of energy metabolism pathways, which should have allowed for elucidating whether E2 actually regulates energy metabolism function in cancer cells. In this last regard, it should be considered that there does not always exist a tight relationship between the mRNA/protein levels with enzyme/transporter activity and metabolic pathway fluxes or biological function (13); the regulatory mechanisms operating at the different levels of biological complexity should also be taken into account for more accurate data interpretation, avoiding unsubstantiated extrapolations.

In the present study, the effect of E2 was systematically analyzed on energy metabolism of breast ER+ cancer cells. Thus. (i) the levels of energy-metabolism associated transcription factors (ERα and β, HIF-1α, c-MYC, p53, PPAR-γ and PGC1- α); (ii) the protein levels and fluxes of glycolysis and oxidative phosphorylation (OxPhos); and (iii) metastatic ATP-dependent processes such as cell invasiveness and migration were determined by using the multicellular tumor spheroids (MCTS) model, a tridimensional cancer cell model (14). MCTS mimic the behavior and structure of cancer cells in their own physiological microenvironment, resembling the solid and non-vascularized initial stages of solid tumors by establishing metabolite gradients including carbon sources, oxygen, H+, added drugs, between peripheral well-oxygenated cell layers, inner poorly oxygenated cells layers and a central necrotic/apoptotic core.

Once the main ATP supplier can be identified in E2-stimulated MCTS, strategies using anti-OxPhos or anti-glycolytic inhibitors could be tested in order to block MCTS growth. This last goal is clinically relevant because the commonly used chemo-therapies (tamoxifen, fulvestran, anti-estrogen analogues, aromatase inhibitors) against ER+ breast cancer do not always provide positive outcomes and such therapies are frequently associated with the development of severe side effects (15–17). This study shall provide the basis for designing improved treatments targeting the principal energy metabolism pathway in hormone-dependent cancers, looking for no adverse side-effects on non-cancer cells functions.

## Materials and methods

### Chemicals

17β-estradiol (E2, Sigma, MO, USA) was dissolved in a mix of ethanol 70%/dimethyl sulfoxide (DMSO) 30%. The maximal amount of ethanol/DMSO used was less than 10% of the final volume in the well, which did not affect the proliferation rate and cellular viability (>95%).

### Cancer cell culture

Human ER+ breast MCF-7 cancer cells (American Type Culture Collection, Rockville, MD, USA) were cultured in Petri dishes in 20 mL of Dulbecco’s Modified Eagle’s Medium (DMEM, Sigma, MO, USA) supplemented with 10% fetal bovine serum (Biowest, Mexico) and 10 000 U penicillin/streptomycin (Sigma-Aldrich, MO, USA). The genotyping (INMEGEN, México) of MCF-7 revealed that the cell line shared 13 from 14 of the canonic allelic markers with the ATCC original clone. For growth and maintenance, cells were incubated in 5% CO_2_/95% air at 37°C and kept until 80-90% of confluence was reached. Then, cells were harvested and use for further experiments (18).

#### Multi-cellular tumor spheroid cultures

For MCTS growth, MCF-7 (1×10^5^ cells/ml) were seeded in 2% (w/v) agarose-coated culture dishes in 5 mL DMEM. After 5 days, old medium was replaced with fresh DMEM in the presence of different E2 (0.1, 1, 10 and 100 nM) concentrations and spheroids were placed under slow orbital shaking (20–50 rpm) at 37°C and 95% air/5% CO_2_. To discard incompletely formed spheroids, fresh DMEM was replaced every three days. The spheroid growth was determined at different culture days by measuring diameters using a calibrated reticule (1/10 mm) in an inverted phase contrast microscope (Zeiss, Thornwood, NY) (14). The growth of each MCTS was followed for 25 days and analyzed by fitting data to the exponential growth curve equation using the Origin 8 software (Northampton MA, USA) (19).

### Western blot

Once maximal size was reached (day 23 of culture), MCTS were recollected and re-suspended in Krebs-Ringer (KR, 125 mM NaCl, 5 mM KCl, 25 mM HEPES, 1 mM KH_2_PO_4_, 1 mM MgCl_2_, 1.4 mM CaCl_2_, pH 7.4) buffer. Samples were centrifuged at 2500 rpm for 3 min, and the pellets were dissolved in RIPA lysis buffer (phosphate buffer saline 1 X pH 7.2, 1% IGEPAL NP40, 0.1% SDS and 0.05% sodium deoxycholate) *plus* 1 mM phenyl methanesulfonyl fluoride (PMSF) and 1 protease inhibitors cocktail tablet (Roche, Mannheimm, Germany). Once the protein concentration was determined by the Lowry method (20), the supernatants were kept at -20°C until use. Samples (50 μg protein) were re-suspended in loading buffer *plus* 5% β-mercaptoethanol, loaded onto 10 or 12.5% polyacrylamide gels and separated under reducing conditions by 10-12% SDS-polyacrylamide gel electrophoresis (21). The proteins were blotted to PVDF membranes (BioRad, Hercules, CA, USA) and Western blot analysis was performed by immunoblotting with the following antibodies. From Novus Biologicals (Littleton, CO, USA), anti-GA (NBPZ-29940). From FineTest (Barcelona, Spain), PGC1-α (NFab06351). From Abcam (Waltham, MA, USA), PYK (ab150377) and PPAR-γ (ab70405). From Santa Cruz Biotechnology (Cambridge, MA, USA) α-tubulin (sc-5286), HIF-1α (sc-13515), ERα (sc-71094), ERβ (sc-53494), c-MYC (sc-40), p53 (sc-101762), GLUT-1 (sc-1603), GLUT-3 (sc-74399), HK-I (sc-46695), HK-II (sc-130358), PFK-1 (sc-31711), LDH-A (sc-130327), PDH (sc-65242), IDH3G (sc-365489), 2-OGDH (sc-49589), ND1 (sc-65237), COX-IV (sc-376731), ATPS (sc-58619) and ANT (sc-11433). All antibodies were used at final dilutions of 1:1000−1:2000. The hybridization bands were revealed with the corresponding secondary antibodies conjugated with horseradish peroxidase (Santa Cruz, MA, USA) and the ECL-plus detection system (Amersham, Buckinghamshire, U.K.). Densitometry analysis was performed using the Scion Image Software (Scion Corp., Frederic, MD, USA) and normalized against α-tubulin, which corresponded to 100% intensity.

### OxPhos and glycolysis fluxes

For assessment of energy metabolism fluxes, MCF-7 spheroids from day 23 of culture were incubated in KR buffer plus trypsin/EDTA (0.25%) for 50 min; afterwards, MCTS were gently and mechanically disaggregated. Cells derived from disaggregated MCTS maintained viability up to 95%.

For glycolysis flux, disaggregated cells (2 mg protein/mL) were incubated in KR buffer. Glycolysis was started by adding 5 mM external glucose (Sigma-Aldrich, MO, USA), and cellular samples were collected after 0 and 10 min of incubation at 37°C under smooth orbital shaking. At the indicated times, the cells were rapidly mixed with 3% (w/v) cold perchloric acid and centrifuged. The supernatants were neutralized with 1N KOH/100 mM Tris. To rule out lactate production by glutaminolysis, cells were also incubated with 2-deoxyglucose (2-DG, 10 mM) (Sigma-Aldrich, MO, USA) (22). Lactate was determined by a standard method with lactate dehydrogenase (Roche, Mannheim, Germany) following the NADH formation at 340 nm (23).

For OxPhos flux, cells (2-5 mg protein/mL) were incubated at 37°C in an air saturated RK medium plus 5 mM glucose. To distinguish between the oxygen consumption by mitochondria (i.e., net OxPhos flux) (22, 24) and non-mitochondrial sources (25, 26), cells were incubated with 5 μM oligomycin (Sigma-Aldrich, MO, USA), a potent, specific, and permeable inhibitor of the mitochondrial ATP synthase (ATPS). The net OxPhos rate was determined by using a Clark type electrode, as previously described (27) and by using a high-resolution respirometer (Oroboros Instruments, Innsbruck, Austria) (22) at 37°C. The contribution of OxPhos and glycolysis to the cellular ATP supply was determined, respectively, from the net OxPhos rate multiplied by the ATP/O or P»/O_2_ ratio that corresponds to 2.5 (28) or 5 (22, 24), and from the rate of lactate production, assuming a stoichiometry of 1 mol of ATP produced per 1 mol of lactate produced (22).

### Cell migration and invasiveness

For cell migration, mature MCF-7 spheroids were disaggregated as described in the previous section. Afterwards, cells were grown in complete DMEM medium in petri dishes (5x10^6^ cells/well) at 37°C and 95% air/5% CO_2_. After reaching 80-90% confluence, cell culture was wounded by using a plastic tip (wound healing assay). Then, culture was washed twice with 37°C PBS (155 mM NaCl, 1.5 mM KH_2_PO_4_, 2.7 mM NaH_2_PO4, pH 7.2) and further incubated with fresh non-serum DMEM. Images of the cellular migration were taken at 0 and 24 h with an inverted microscope (Zeiss, Thornwood, NY, USA). For each experiment, the cellular migration distance from the border to the center of the petri dish was measured with a graduated reticule (Zeiss, Thornwood, NY, USA) (18).

For invasiveness assays, cells from disaggregated MCTS were incubated in free-serum DMEM for 24 h at 37°C and 95% air/5% CO_2_. Afterwards, the cells were washed, re-suspended in free-serum DMEM medium, and placed in the upper compartment of 96-multiwell Boyden chambers (Merck Millipore, MA, USA) at a final concentration of 5×104 cells/well; the Boyden chamber lower compartment was filled with free-serum DMEM. Then, the Boyden chamber was incubated at 37°C and 95% air/5% CO_2_ for 24 h. To detect invasive cells in the lower chamber compartment, cells were incubated with 60 nM calcein-AM for 1 h at 37°C and calcein-fluorescence was detected at 485 nm excitation and 520 nm emission by using a microplate reader (Nunclon, Roskilde, Denmark) (18).

### Determination of drug IC_50_ (concentration required to reach 50% inhibition) values in MCF-7 MCTS

For evaluation of drug effect on MCTS growth, MCF-7 (1×10^5^ cells/mL) were seeded in 2% (w/v) agarose-coated culture dishes in 5 mL DMEM. After 5 days, old medium was replaced with fresh DMEM in the presence of E2 (0, 10 and 100 nM) and either canonical anti-cancer (DOXO, doxorubicin), anti-glycolytic (IOA, iodoacetate) or anti-mitochondrial (CXB, celecoxib) drugs at 1, 10, 100, 250 and 500 nM concentrations (29). Afterwards, MCTS were placed under slow orbital shaking (20-50 rpm) at 37°C and 95% air/5% CO_2._ The MCTS growth in the presence of each inhibitor was determined at different culture days by measuring diameters using a calibrated reticule (1/10 mm) in an inverted phase contrast microscope (Zeiss, Thornwood, NY) (14). The IC_50_ inhibitor value for MCTS growth was determined at day 23 of culture corresponding to the time in which the MCTS maximal size was reached.

### Data analysis

Experiments were performed with at least three independent cell preparations (n) (30). The data shown represent mean ± standard deviation (S.D.). Student’s *t* test and ANOVA/*post hoc* Scheffe (31, 32) analyses with P values < 0.05 or lower were used to determine statistical significance.

## Results

The results presented in this study were performed in the human ER+-breast MCF-7, the most common cancer cell line used as experimental model of ER-positive breast cancer, because these cells closely resemble several characteristics (*i.e.*, cellular phenotype, *in vivo* morphology, drug-resistance) found in patients with ER-positive breast tumors (33, 34).

### Effect of 17β- estradiol (E2) on MCF-7 MCTS growth

**Figure 1:**
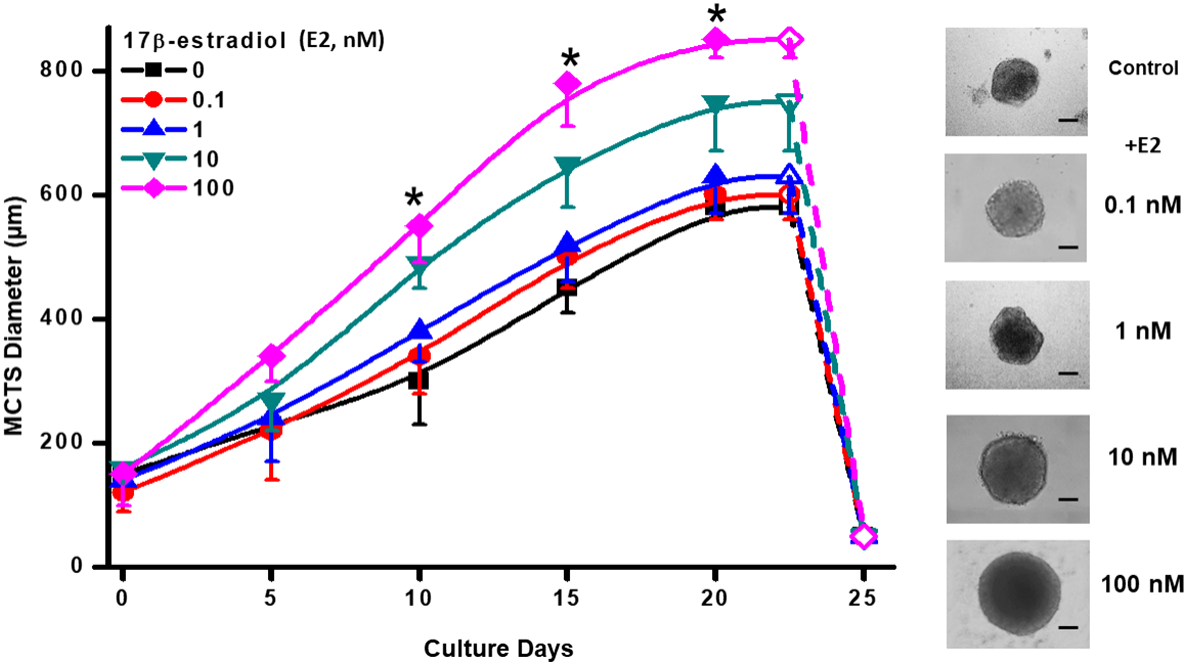
Effect of E2 on MCF-7 MCTS growth. The right panel shows contrast phase microscope images taken at day 20, which display the typical different MCTS sizes in the presence of exogenous E2. Scale Bar= 200 µm. Data shown represent the mean ± S.D. of at least 3 different independent preparations, n=30-40 spheroids. *P <0.05 vs. 10 and 100 nM of E2. E2, 17β-estradiol.

Human ER+-breast MCF-7 MCTS reached a maximal spheroid diameter of 580 ± 20 nm at day 23 of culture (**Figure 1**) as it was previously reported (35). The presence of E2 at physiological concentrations of 10 and 100 nM (34) increased MCTS diameter by 29% (750 ± 80, n= 30 spheroids) and 46% (850 ± 50, n= 30 spheroids), respectively at day 23 (**Figure 1**). Non-physiological and lower E2 concentrations (0.1 or 1 nM) were not able to promote MCTS growth throughout the culture time assessed (**Figure 1**). Morphology and maximal diameter of MCF-7 MCTS treated with 10 or 100 nM E2 was like that reported for other large-size tumor spheroids (14). After day 23 of culture, MCF-7 MCTS become unstable and spontaneously disaggregate (19). Although E2 stimulated MCTS growth, it was not able to prevent spontaneous and fast MCTS disaggregation after day 24 of culture (**Figure 1**). Because MCF-7 MCTS growth was stimulated with 10 and 100 nM E2, subsequent experimentation was conducted by using these hormone concentrations.

### Estrogen receptors (ER) and ER-target protein levels in MCF-7 MCTS exposed to 17β-estradiol

**Figure 2:**
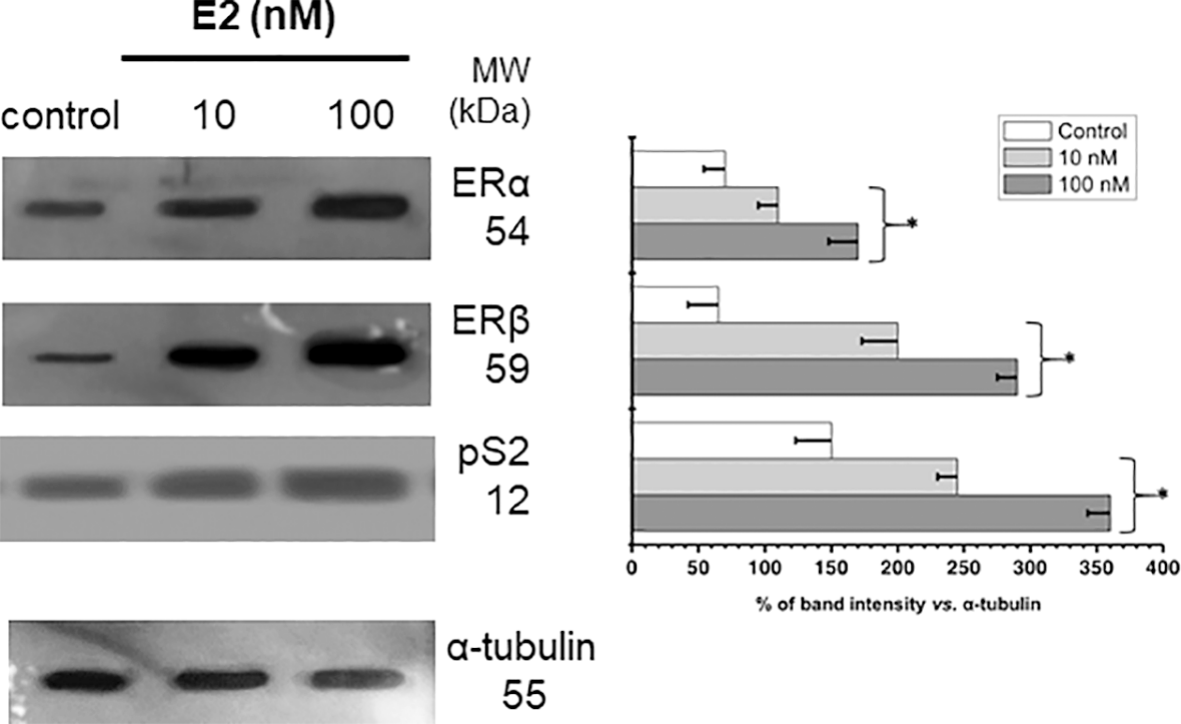
Effect of E2 on the contents of ERs and PS2 in MCF-7 MCTS. Histograms represent a double normalization against α-tubulin. Data shown represent the mean ± S.D. of at least three different preparations. * P <0.05 vs. control (non-treated cells).

MCF-7 MCTS incubation with 10 or 100 nM E2 promoted a significant increment in the level of E2 receptors ERα (1.6-2.4 times) and ERβ (3.1-4.5 times) *vs*. non-hormone incubated MCTS (**Figure 2**). Change in ERα and ERβ contents correlated with an elevation by 60-140% in the well-known E2-target pS2, a polypeptide with growth factor presumed function (3, 36, 37), supporting functional status for both α and β E2 receptors (**Figure 2**).

### Energy associated-transcription factors and energy metabolism proteins levels in MCF-7 MCTS exposed to 17β-estradiol (E2)

**Figure 3:**
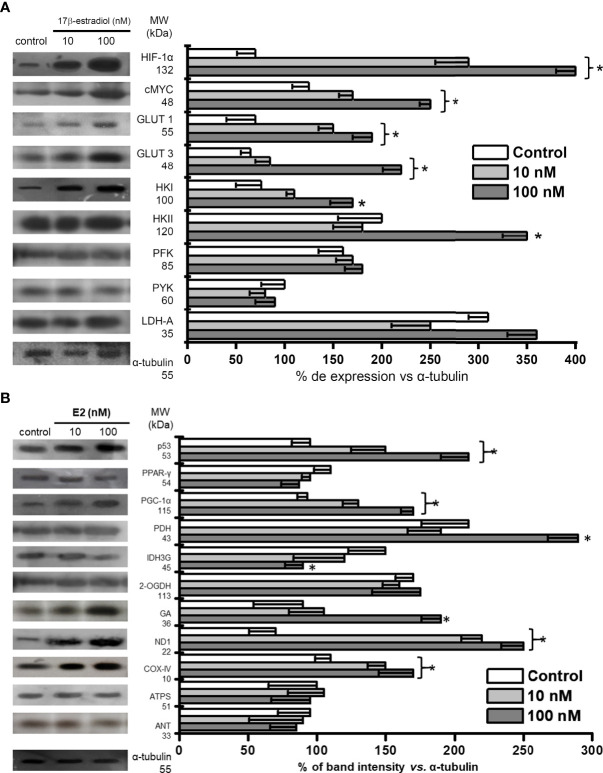
Effect of E2 on the contents of **(A)** transcription regulators and protein contents involved in glycolysis pathway; **(B)** transcription regulators and protein contents involved in OxPhos pathway of MCF-7 MCTS. Histograms represent a double normalization against α-tubulin. Data shown represent the mean ± S.D. of at least three different preparations. * P <0.05 vs. control (non-treated cells). The numerical values on the left y-axis indicate the respective molecular weights.

The increased ERα and ERβ levels induced by E2 correlated with significant increments in the glycolytic transcriptional modulators HIF1-α (3.4-5.7 times) and c-MYC (1.4-2 times) *vs*. non-hormone treated MCTS (**Figure 3A**). Because of the elevated HIF1-α and c-MYC levels, their glycolytic targets GLUT-1, GLUT-3, HK-I, and HK-II were also increased by 1.8-3.4 times *vs*. non-treated cells (**Figure 3A**). The levels of other glycolytic proteins like PFK-1, PYK and LDH-A remained without change.

The mitochondrial biogenesis-associated transcription regulator PGC1-α level was also increased 1.4-1.8 times by E2 (**Figure 3B**). No effect was observed on the level of PPAR-γ, which is involved in fatty acid storage and glucose metabolism (38). In addition, E2 increased the level of some respiratory chain proteins like ND1 (complex I) and COX-IV (complex IV) by 1.4-3.6 times vs. non-treated MCTS. Increments in PDH (1.4 times) and GA (2.1 times) levels were observed at E2 100 nM. Hormone did not affect the content of 2-OGDH, ATPS or ANT, but promoted a significant diminution (40%) in the Krebs cycle IDH3G level (**Figure 3B**).

### Effect of 17β- estradiol (E2) on MCF-7 MCTS energy metabolism fluxes

**Figure 4:**
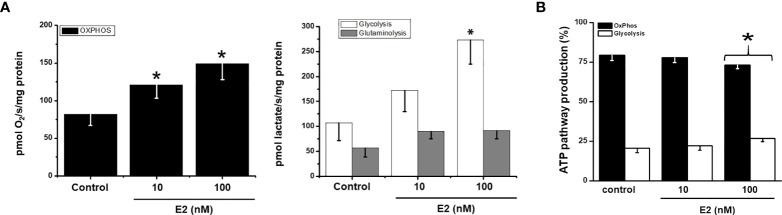
Effect of E2 on **(A)** energy metabolism fluxes and **(B)** ATP supply from glycolysis and OxPhos in MCF7 MCTS For OxPhos, flux represents the rate of oligomycin-sensitive oxygen consumption; for glycolysis, flux represents the rate of 2DG-sensitive lactate production; for glutaminolysis, flux represents the rate of 2DG-resistant lactate production. For ATP supply from OxPhos an ATP/O ratio of 2.5 (28), or P»/O2 ratio of 5 (22, 24), was used; for ATP supply from glycolysis it was assumed a stoichiometry of 1 mol of ATP produced per 1 mol of lactate formed. Data shown represent the mean ± S.D. of at least three different preparations. * P <0.05 vs. control (non-treated cells).

Both glycolysis (61-156%) and OxPhos (48-83%) fluxes were stimulated by 10 and 100 nM E2 (**Figure 4A**) correlating with a rise of several mitochondrial and glycolytic protein levels (**Figures 3A, B**). Glutamine oxidation (i.e., glutaminolysis) also increased by 60% further supporting E2-induced OxPhos up-regulation.

In the absence of E2, MCF-7 MCTS showed OxPhos as the predominant ATP supplier. Although both energy pathways were stimulated by E2 (**Figure 4A**), OxPhos remained as the principal energy provider (>75%) to sustain cancer cell processes (**Figure 4B**) (18).

### Effect of 17β-estradiol (E2) on MCF7 MCTS cancer EMT, migration and invasiveness

Tridimensional architecture of MCTS favors the development of metastatic phenotype (21, 35). Thus, epithelial mesenchymal transition (EMT) marker proteins (SNAIL, fibronectin, E-cadherin, MMP-1, MMP-9 and vimentin) as well as cancer cell migration and invasiveness processes were analyzed in the presence of E2.

**Figure 5:**
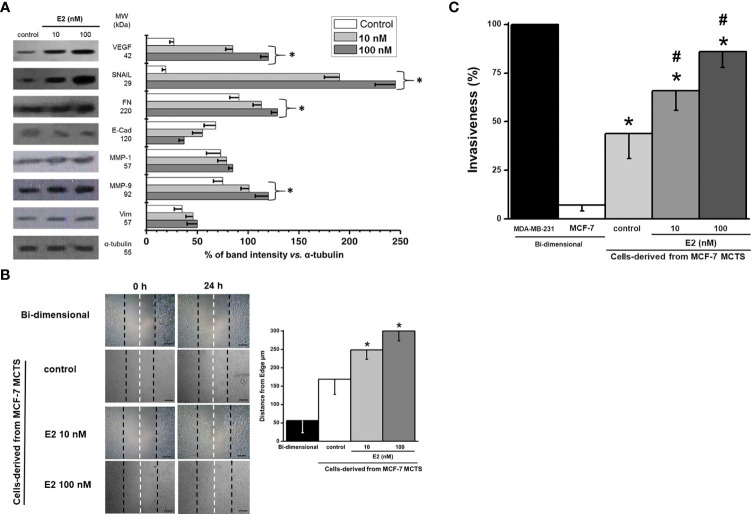
Effect of E2 on **(A)** levels of EMT proteins of MCF-7 MCTS; **(B)** migration; and **(C)** invasion of cells derived from MCF-7 MCTS. For cell migration, contrast phase microscope images were taken at the beginning (t=0) and after 24 h E2 exposure, scale bar= 200 µm; right panel represents the distance traveled and calculated from the edge to the center of cell dish. Invasiveness was assessed by using multiwell Boyden chambers as it was indicated in the Material and Methods section. Data shown represent the mean ± S.D. of at least three different preparations. * P <0.05 vs. control (non-treated cells).

The stimulation of both metastatic processes (migration and invasiveness) by E2 correlated with a significant increase in several proteins related with (i) epithelial-mesenchymal transition (EMT) like SNAIL (7-10 times) and VEGF (2.7-4 times); (ii) extracellular matrix degradation proteins like MMP-1 (8-16%) and MMP-9 (34-60%); and (iii) motility associated proteins like vimentin (30-40%) and fibronectin (24-40%) (**Figure 5A**). In addition, the cell adhesion protein E-cadherin decreased by 19-45% (**Figure 5A**).

The cells from disaggregated MCF-7 MCTS maintained a high migratory capacity vs. their parental MCF-7 monolayer cells (**Figure 5B**). E2 (10 and 100 nM) increased cell migration by 30 and 78%, respectively (**Figure 5B**).

Other ATP-dependent process like cellular invasion was also analyzed in the presence of E2 (**Figure 5C**). The well-known highly invasive breast cancer MDA-MB-231 cell line was used as reference control to assess the relative invasiveness capacities of MCF-7 MCTS cells exposed to E2. As expected, bi-dimensional MCF-7 cells showed negligible invasiveness capacity (<10% vs. MDA-MB-231) confirming their low metastatic phenotype (39) (**Figure 5C**). In contrast, MCF-7 cells derived from disaggregated MCF-7 MCTS developed invasion capacity (35), which was significantly stimulated (50-95%) by E2.

### Dependence of cell invasion and migration on OxPhos ATP supply in 17β-estradiol (E2) stimulated MCF-7 MCTS

**Figure 6:**
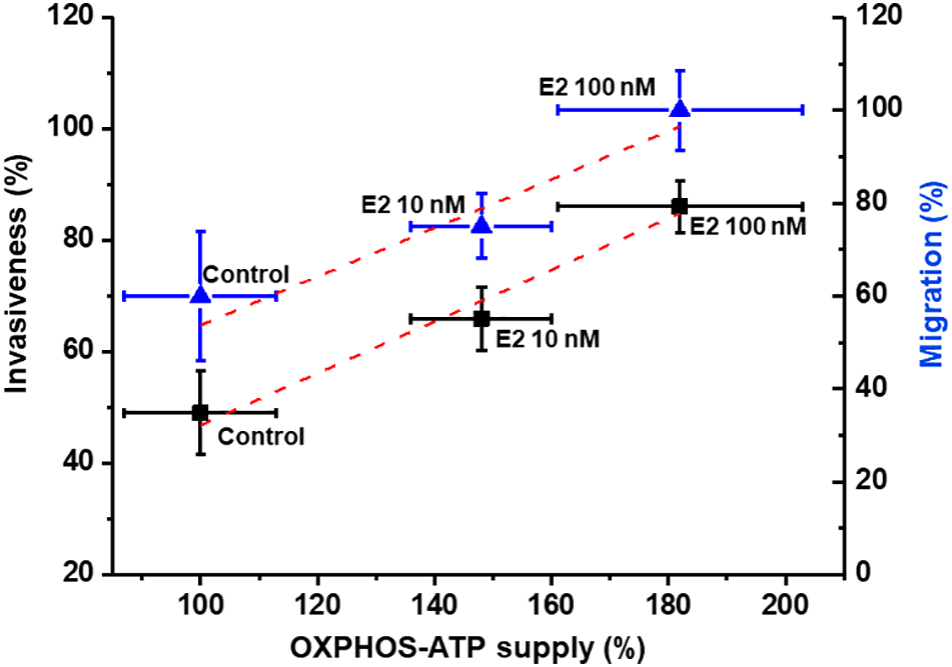
Dependence of invasion and migration on OxPhos ATP supply of cells derived from MCF-7 MCTS. Net OxPhos flux and metastasis processes were determined in the presence of E2 as described in the Material and Methods section. For cell invasion and migration, the 100 % value for net OxPhos flux without E2 was 408 ± 55 pmol O2/s/mg cell protein for cells derived from MCF-7 MCTS. For cell migration, the 100 % value was 290 ± 20 µm displacement for cells derived from MCF-7 MCTS after 24 h. Data represents the mean ± error standard of at least three different preparations.

The stimulation of MCF-7 MCTS energy pathways by E2 (10 or 100 nM) prompted that the intracellular ATP supplied by OxPhos remaining at prominent level (**Figure 4B**). It has been suggested that several biological functions in cancer cells mostly depend on the ATP provided by energy metabolism pathways (40, 41); but this assumption has not been experimentally demonstrated as yet. Therefore, to establish the dependence of cancer cell invasion and migration on net OxPhos ATP supply (**Figure 6**) or net cellular (OxPhos + glycolysis) ATP supply (**Figure S1**), the ATP supply derived from OxPhos or from the sum of both energy pathways was plotted vs. cellular migration and invasiveness.

As the rate of ATP synthesis increases as result of E2-OxPhos pathway stimulation, a proportional increase in both migration and invasiveness was observed, demonstrating a strict relationship between metastatic processes and the ATP supply derived from E2-stimulated OxPhos. Similar relationships were found when metastatic processes were plotted vs. total cytosolic + mitochondrial ATP supply. Under these conditions and applying metabolic control analysis (42, 43), the flux control coefficients were calculated from the initial slope of the plots shown in **Figure 6**, starting at the 100% reference control point, which corresponds to non-added E2 (control condition). Thus, it was established that OxPhos pathway exerted high control of about 0.5 on both cancer cell migration and invasiveness after E2 treatment, i.e., the metastatic processes depended 50% on OxPhos (**Figure 6**). Total ATP supply (glycolysis + OxPhos) exerted a similar control on the metastasic processes (**Figure S1**).

### Effect of OxPhos inhibition on growth and ATP dependent-processes in 17β-estradiol (E2) stimulated MCF-7 MCTS

**Figure 7:**
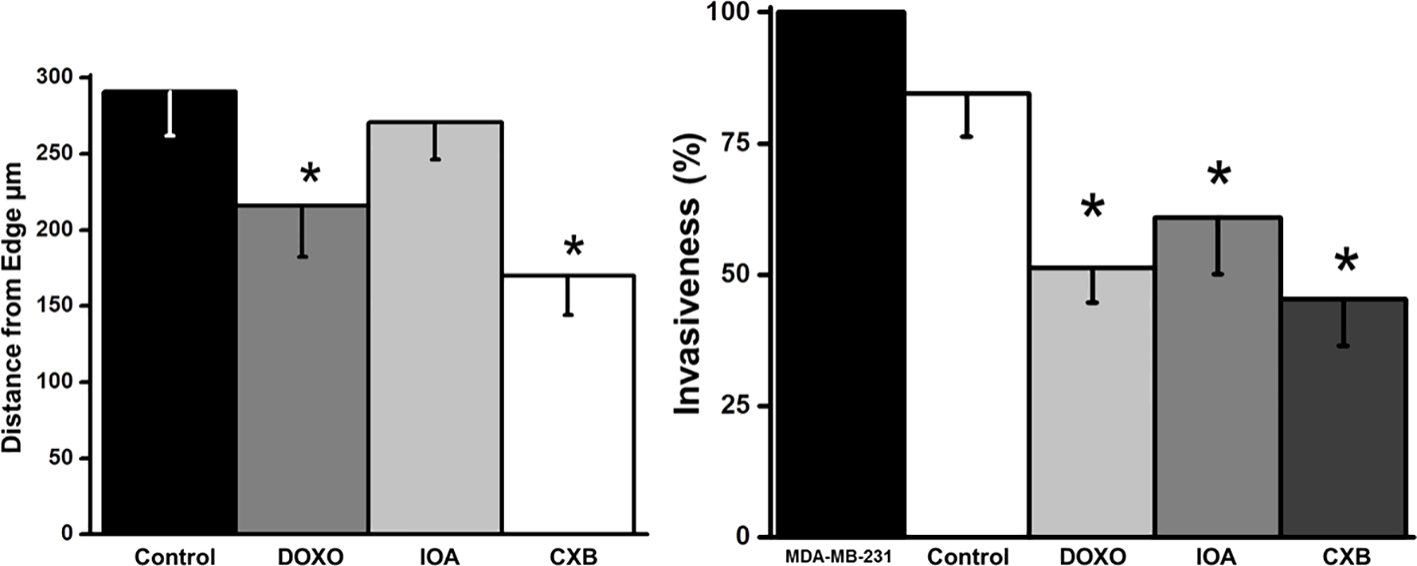
Effect of celecoxib (CXB), doxorubicin (DOXO) and iodoacetate (IOA) on migration and invasiveness of cells derived from MCF-7 MCTS. Drugs were added as it was indicated in the Material and Methods section at IC50 concentrations reported in **Table 1**. Data shown represent the mean ± S.D. of at least three different preparations. *P <0.05 vs. control (non-treated cells).

OxPhos dependence of cellular migration and invasiveness were also analyzed in complete and intact MCF-7 MCTS. Celecoxib (CXB) is a repurposed NSAID, which displays strong inhibitory effect on OxPhos and growth of several metastatic cancer cell lines (29, 44). CXB at nanomolar doses decreased the growth of MCF-7 MCTS (IC50 = 5 nM); same CXB doses blocked the E2-stimulated OxPhos by 60% in cells derived from MCF-7 MCTS (data not shown). Likewise, CXB blocked E2-stimulated MCF-7 MCTS growth at low nanomolar concentrations (**Table 1**). CXB at IC50 concentrations for decreasing MCTS growth, potently inhibited both invasiveness (46 ± 7.5%) and migration (41 ± 5%) (**Figure 7**). On the contrary, glycolytic inhibitors such as (iodoacetate) at concentrations at which glycolysis is inhibited by 65% or canonical anticancer drugs like doxorubicin required higher drug concentrations (50-500 nM) to slightly affect E2-stimulated MCF-7 MCTS growth and metastasis (**Table 1**; **Figure 7**).

**Table 1:** IC_50_ values (nM) of metabolic and canonical drugs for MCF-7 MCTS growth.

	Control	E2 (nM)	
		10	100
DOXO	38 ± 8	47 ± 10	72 ± 9
IOA	71 ± 9	178 ± 10	>500
CXB	5 ± 0.7	8 ± 0.6	10 ± 1

Drugs were added at the beginning of MCTS formation as it was indicated in the Material and Methods section. Data shown represent the mean ± S.D. of at least 3 different preparations; n=30-40 spheroids. Control, non E2 added. DOXO, doxorubicin; IOA, iodoacetate; CXB, celecoxib.

## Discussion

The substantial majority of studies showing the effect of E2 on cancer cell proliferation (45, 46), survival, angiogenesis (47), migration, invasion (48) or metastasis (17) have been performed in bi-dimensional cell cultures. Although bi-dimensional models have been helpful for understanding the biochemistry and physiology of cancer cells, such information has been difficult to translate into treatment and early detection or biomarker identification of actual tumors, because bi-dimensional cultures does not reflect the physiological behavior of tumor cells *in situ*. Therefore, the use of multicellular tumor spheroids (MCTS) emerges as a valuable tool for the identification of metabolic changes in solid tumors exposed to several factors (i.e., sexual hormones, chemotherapies). The different micro-regions found in large, mature MCTS lead to the development of cells with different phenotypes, as occurs in solid tumors, located at different distances from the blood nourishing capillaries (49, 50). Thus, more closely realistic responses to E2 may be found in MCTS compared to bi-dimensional culture cells, which may allow envisioning better strategies for the treatment of Her2+-breast tumors.

### 17β-estradiol (E2) decreased the Erα/Erβ ratio in ER+ breast cancer MCTS

MCF-7 cells in tridimensional culture displayed high sensitivity to E2 for growth and ERα, ERβ and PS2 protein levels. Both ERs were increased by E2, with the levels of ERβ being higher than those of ERα. In this regard, there are some reports indicating that imbalance of these two estrogen receptors may lead to the occurrence of aggressive breast cancers. For example, it has been documented that ERβ downregulation prevents breast cancer cell migration through tyrosine kinase receptor [epidermal growth factor receptor (EGFR)/insulin-like growth factor-I receptor (IGF-IR)] and Janus kinase/signal transducer and activator of transcription (JAK/STAT) signaling pathways [reviewed by (51)].

### 17β-estradiol (E2) enhances ER+ breast MCTS growth

Physiological E2 concentrations stimulated by 29-46% the growth of MCF-7 MCTS. Similar effects of E2 have been observed in other studies using MELN (cells derived from MCF-7 cells) spheroids (52). E2 also stimulated MCF-7 cell proliferation in bi-dimensional cultures (46). The increase in cyclin D1 mRNA content *via* c-Jun/AF-2 signaling pathway (45); the suppression of p53/p21; and the up-regulation of PCNA and Ki-67 (46) are molecular mechanisms proposed to explain how estradiol may stimulate MCF-7 cell growth in bi-dimensional cultures. On the contrary, in non-cancer cells E2 blocks proliferation acting as a potent suppressor of Janus kinase pathway/cytokine signaling-2 protein involved in growth hormone activation (53); as well as inducing the release of E2-regulated autocrine growth factors, which antagonize and block the growth factor receptors [reviewed by (54)], thus limiting cellular proliferation.

Scarce information is available concerning E2 -dependent regulation of the cell cycle machinery in tridimensional models that resemble solid tumor physiology. Truchet et al. (52), showed in MELN (cells derived from MCF-7 cells) spheroids that E2 (1 nM, from 4 to 23 days incubation) induced a proliferation proteins gradient from MCTS periphery (higher content of Ki67- and cyclinD1- positive cells) to the center (low content of Ki67- and cyclin D1-positive cells), which may help explaining why E2 stimulates cell cycle in cancer spheroids. In the present study, the up regulation of energy metabolism pathways by E2 for ATP supply was demonstrated and it is proposed as an essential molecular mechanism, which together with the well-known effect of E2 on cell cycle, modulates solid tumor growth.

### 17β-estradiol (E2) stimulates glycolytic metabolism in MCF-7 MCTS

The tridimensional arrangement of tumor spheroids leads to the development of several cell layers with important, marked metabolic differences (14). The inner center core of large spheroids becomes hypoxic and necrotic, promoting HIF-1α stabilization and glycolysis activation (c.f. **Figure 3A**) (14, 55). In MCTS, the level of HIF-1α was significantly increased (>3 times) by E2, as a consequence of estrogen-response elements in the HIF-1α gene (56); and direct interaction of ERα with HIF-1α (57).

Derived from HIF-1α stabilization, several HIF1α-induced glycolytic proteins are overexpressed (55) like the flux-controlling steps (58) GLUT-1, GLUT-3, HK-I and HK-II (c.f. **Figure 3A**) detected in E2-exposed MCF-7 MCTS vs. non-treated MCTS. Indeed, this last result correlates with some observations found in bi-dimensional culture cancer (hepatocellular carcinoma HepG2, MCF-7, T47D) cells incubated with E2 (1-100 nM). In these last models, E2 promotes (i) mRNA increase of glycolytic genes (GLUT-2, PFK-1) (10, 59); (ii) overexpression of glycolytic pathway genes through E2-induced PI3K/AKT activation (60); (iii) increased levels of the main PFK-1 co-activator Fru2,6BP (10); and (iv) increased activity of mitochondrial bound-HK (61).

Other observations showing up-regulation of glycolysis by estradiol include the stimulation of glucose uptake in bi-dimensional breast T47D and MCF-7, and cervix HeLa and SiHa cancer cells (10, 61, 62). E2 (10 and 100 nM) increased the glycolytic flux >45% in MCF-7 MCTS vs. non treated cells. This increment in glycolysis resembled the elevated lactate release to the extracellular milieu reported in MCF-7, T47D, HeLa and SiHa cells exposed to E2 (10 nM/24 h or 1 week) (61–63).

In a human biopsy study including 88 human breast Her2+ (luminal A and luminal B) cancer samples, it was found that the ERβ but not ERα isoform predominates in the stem cell phenotype. ERβ stimulation with diarylproprionitrile, its selective ligand, increased mammosphere growth as well as glycolytic metabolism, increasing the mRNA level of several genes related with glucose metabolism (HIF-1α, n-MYC, GLUT-1, PFK/FBP-4, glycogen branching enzyme GBE1), and glycolytic flux (64).

### 17β-estradiol (E2) stimulates OxPhos pathway in MCF-7 MCTS

The lipophilic property of E2 allows for passive diffusion into mitochondrial membranes from the external culture medium. Moreover, E2 may be rapidly delivery into mitochondria *via* receptor-mediated endocytosis in hepatocarcinoma HepG2 cells (65).

In MCF-7 cells, a substantial fraction (approximately 20%) of total cellular ER is localized within the mitochondrial matrix (66). In addition, it has been documented that ERα and ERβ interactions with E2 improves mitochondrial function acting at different levels. These actions include (a) favoring a large and clear mitochondrial morphology (67); (b) activating transcription factors containing E2-response elements such as the Nuclear Respiratory Factor 1 (NRF1) (12) involved in the overexpression of mitochondrial metabolism (i.e. COX, ND1, SDH, bc-1complex, ATPS) enzymes (68), and biogenesis (12) genes, some of them correlating with elevated protein level (COX-IV) (12, 69); (c) activating TFAM, a NRF-1 target, which binds and promote mtDNA transcription of genes encoding for mitochondrial respiratory complex proteins (70).

Unfortunately, in all above-mentioned studies where E2 has shown an up-regulation effect on OxPhos, solely the transcript levels have been evaluated but proper mitochondrial functions have not been analyzed. This takes relevance because not always the transcript levels correlate with the related protein content and activity. Moreover, not often mRNA levels correlate with changes in metabolite intermediaries and pathway fluxes, or biological function, since robust regulation mechanisms are in place at the distinct levels of biological complexity (13).

Other studies have revealed contrasting results to those described above. For instance, Sastre-Serra et al. (71), found that E2 (1 nM, 48 h) diminished the TFAM and COX-IV levels and the activities of mitochondrial enzymes like CS, COX and ATP synthase in bi-dimensional culture MCF-7 cells. It was argued that low mitochondrial functionality of MCF-7 cells in the presence of E2 was linked to the presence of a high ERα/ERβ ratio (71). Hence, the tridimensional architecture of MCF-7 promoting a low ERα/ERβ ratio (c.f. **Figure 2**) could then favors mitochondrial metabolism.

In MCTS, the content of several mitochondrial proteins (COX-IV) significantly increased in the presence of E2 like reported for bi-dimensional MCF-7 cells (12, 69). However, the content of other OxPhos related proteins in 2-D cultured cancer cells, except for the study by Sastre-Serra et al. (71), has not been analyzed, and thus for comparative purposes, information is not available. In non-cancer cells (rat liver, brain, heart), E2 clearly promotes significant lowering in the content of OxPhos proteins (complex I, II, III, IV, and ATPS) (72, 73). Regarding OxPhos pathway flux, scarce (bi-dimensional cultures) or null (spheroids/mammospheres) information about E2 effects is available for Her2+ breast cancer cells. Radde et al. (74), observed negligible activation (less 5%) in the ATP-linked oxygen consumption of MCF-7 cells exposed to E2 (24 h, 1 nM, 10 nM). However, under serum starvation, the ATP-linked oxygen consumption and the maximal respiratory capacity of MCF-7 cells was stimulated (40-70%) by E2.

This last observation concurred with the strong OxPhos stimulation by E2 observed in MCF-7 MCTS (c.f. **Figure 3A**), further suggesting that serum components with limited availability due to the tridimensional architecture of spheroids were also involved in the OxPhos activation by E2. In addition, it is noted that data from bi-dimensional ER+ cancer cells [10, 59-63, 67-70], including results from the present study, support the conclusion that estradiol also potently promotes glycolytic and OxPhos activation in 3D-rearregment cancer cell models.

### 17β-estradiol (E2) enhances metastatic phenotype in MCF-7 MCTS

The epithelial–mesenchymal transition (EMT) promotes stationary cancer cells to migrate and invade. Indeed, E2 induced that MCF-7 MCTS-derived cells overexpressed several EMT proteins like SNAIL, FN, MMP-9 and Vim and decreased E-cadherin level (c.f. **Figure 5A**). In these cells, E2 (5 nM/72h) also induced loss of epithelial cell polarity, separation into individual cells, subsequent dispersion after the acquisition of cell motility, and loss of E-cadherin, indicating the development of mesenchymal cell-like characteristics. Tamoxifen prevented EMT process (75), suggesting that the E2/ER axis is involved in EMT activation.

MCF-7 MCTS showed an increased migration and invasiveness capacity induced by E2 (c.f. **Figures 5B, C**). Similarly, several studies in bi-dimensional breast and glioma cancer cells show that E2 (1-100 nM/24 h) stimulates cell migration. This is accomplished by inducing (i) the activation of signaling proteins (c-Src, FAK, paxillin, ERK and protein kinase B phosphorylation) related with cell migration (76); (ii) binding to a G protein coupled to ER (GPR30); this GPR30/E2 complex triggers a protein kinase (PRKACB) to phosphorylate and inactivate the aquaporin AQP2, which is involved in cancer cell migration (77). On the other hand, the strong HIF-1α stabilization observed in E2-stimulated MCF-7 MCTS may up-regulate angiogenic and migratory target genes such as that encoding for VEGF (57).

Furthermore, E2 (10-100 nM) in MCF-7 bi-dimensional cultures promotes cell-cell adhesion (78) and favors tumor formation in nude mouse (79, 80). This last result seems in consonance with our observation that E2 promotes spheroids formation (c.f. **Figure 1**). However, there are not reports indicating that cancer cells derived from spheroids develop mechanisms to increase their ability to invade. E2 enhances the phosphorylation and activation of ezrin, a protein related with the activation of PI3K/Akt/ROCK-2 signaling cascade, which promotes horizontal T47-D cell migration and invasion in three-dimensional matrices (81).

### OxPhos inhibition for blocking 17β-estradiol-dependent aggressive tumors

The present study demonstrates for the first time that estradiol preserves that MCF-7 MCTS be predominantly oxidative. Both energy metabolism pathway fluxes in MCF-7 MCTS were rigorously assessed by correcting lactate production (glycolysis, 2DG-sensitive lactate production) derived from glutaminolysis, as well as total cellular oxygen consumption (OxPhos, oligomycin-sensitive respiration) derived from extramitochondrial sources and passive H+ leak across the inner mitochondrial membrane (22). Although in other studies, glycolysis and/or oxygen consumption fluxes were analyzed in the presence of E2, the contribution to ATP supply from each energy pathway was not determined.

Metabolic therapy targeting cancer mitochondria can be a promising alternative treatment. Our results (c.f. **Figure 4B**) clearly indicated that the principal ATP supplier in cancer cells was OxPhos pathway. In addition, cellular migration and invasiveness depended on ATP-derived from OxPhos with flux control coefficients of about 0.5, but not on glycolysis (c.f. **Figures 6**, **S1**) (18). Thus, both metastatic processes were further titrated with the alternative OxPhos inhibitor celecoxib (CXB), a repurposed NSAID, and its effect was compared with the glycolytic inhibitor iodoacetate, and with doxorubicin, a canonical anti-cancer drug. Up to 10 µM CXB has several targets including cyclooxygenase-2 inhibition (82); and apoptosis activation (83). Recently, it was demonstrated that at low doses CXB blocked OxPhos flux affecting mitochondrial membrane potential (18, 44) and consequently, cancer cell growth was severely impaired (44). For doxorubicin and iodoacetate, high doses were required to affect MCTS growth. Therefore, CXB may be considered as a suitable and promising therapeutic drug for the inhibition of estradiol dependent cancer growth.

## Conclusion

The data of the present study show for the first time that E2 promotes marked increase in growth, energy metabolism and metastatic processes of MCTS. The data also showed a strong dependence of metastatic processes (i.e., invasiveness and migration) on the ATP derived from OxPhos. Therefore, the application of cancer chemotherapies based on OxPhos targeting by using repurposed drugs like celecoxib may help in the clinical setting against hormone-dependent tumors. The present results provide support for modifying and perhaps improving clinical strategies by using re-purposed drugs such as NSAIDs as adjuvant therapy. For cervical cancer cells, CXB has showed significant efficacy in combination with canonical chemotherapy drugs such as cisplatin, paclitaxel or doxorubicin (29). These drug combinations might be useful for the specific treatment of estradiol-dependent cancers.

## Data availability statement

The raw data supporting the conclusions of this article will be made available by the authors, without undue reservation.

## Author contributions

SR-E, SP-V: study conception and supervision, experimental design, manuscript writing, manuscript proofreading, manuscript revision. SP-V, IO-M, JV-N, JP-F, DR-C, GT-M, JG-R: methodology, data acquisition. SP-V, RM-S, SR-E: data analysis. SP-V, statistical analysis. IP-C, JA-P, RM-S, SR-E: manuscript proofreading and revision. All authors contributed to the article and approved the submitted version.

## Funding

The present work was partially supported by grants from CONACyT-México to SCPV (No. 377873), SRE (No. 283144) and RMS (No. 6379); and from PAPIIT, DGAPA-UNAM to SRE (No. IA201823).

## Conflict of interest

The authors declare that the research was conducted in the absence of any commercial or financial relationships that could be construed as a potential conflict of interest.

## Publisher’s note

All claims expressed in this article are solely those of the authors and do not necessarily represent those of their affiliated organizations, or those of the publisher, the editors and the reviewers. Any product that may be evaluated in this article, or claim that may be made by its manufacturer, is not guaranteed or endorsed by the publisher.

## Abbreviations

ANT, adenine nucleotide translocase; ATPS, ATP synthase; COX-IV, cytochrome c oxidase; ERα, estrogen receptor alpha; ERβ, estrogen receptor beta; E2, 17β-estradiol; E-Cad, E-cadherin; FM, fibronectin; GA-L, glutaminase L; GLUT-1, glucose transporter 1; GLUT-3, glucose transporter 3; HIF-1α, hypoxia-inducible factor 1-alpha; HK-I, hexokinase I; HK-II, hexokinase II; IDH, isocitrate dehydrogenase; LDH-A, lactate dehydrogenase A; MCTS; multicellular tumor spheroids; MMP-1, matrix metallopeptidase 1; 9MMP-9, matrix metallopeptidase 9; ND1, NADH dehydrogenase subunit 1; NSAID, non-steroidal anti-inflammatory drug; OxPhos, oxidative phosphorylation; PDH, pyruvate dehydrogenase; PFK-1, phosphofructokinase 1; PGC1-α, peroxisome proliferator-activated receptor gamma coactivator 1-alpha; PPAR-γ, peroxisome proliferator-activated receptor gamma; PYK, pyruvate kinase; Vim, vimentin; 2DG, 2-deoxyglucose; 2-OGDH, 2-oxoglutarate dehydrogenase.
