# Supplementary material for: 17-β Estradiol up-regulates energy metabolic pathways, cellular proliferation and tumor invasiveness in ER+ breast cancer spheroids
Source: Front Oncol. 2022 Nov 7;12:1018137. doi: 10.3389/fonc.2022.1018137 (PMC9676491; doi:10.3389/fonc.2022.1018137)
Supplement: Supplementary file 1 [file Image_1.pdf]

frontiers

Supplementary Material

1.1 Supplementary Figures

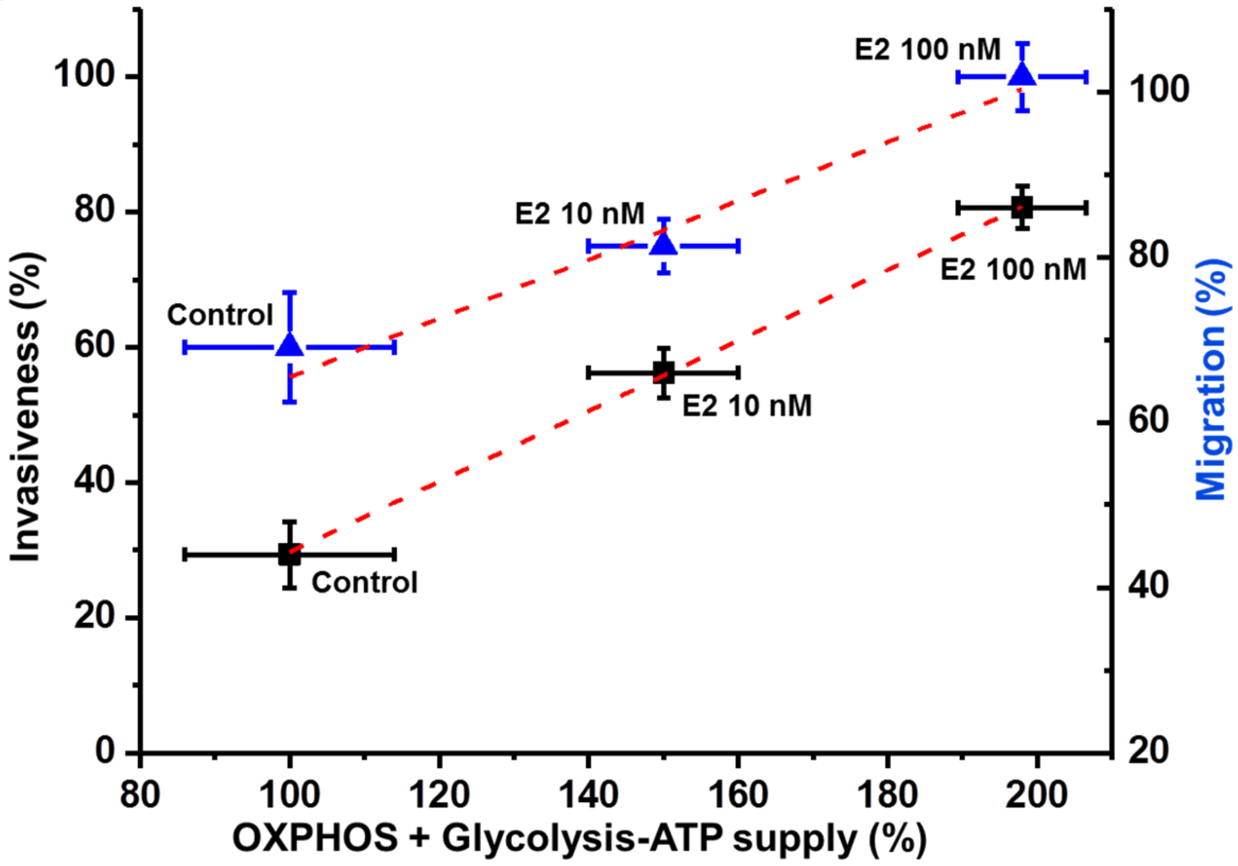

Supplementary Figure 1. Dependence of cancer cell invasion and migration on OXPHOS plus Glycolysis ATP supply of cells derived from MCF-7 MCTS. Net OXPHOS flux and metastasis processes were determined in the presence of E2 as it is described in the Material and Methods section. For cell invasion and migration, the 100 % value for net ATP supply without E2 was 515 ± 72 pmol ATP/s/mg cell protein for cells derived from MCF-7 MCTS. For cell migration, the 100 % value was 291 ± 25 µm displacement for cells derived from MCF-7 MCTS after 24 h. The values represent the mean ± standard error.
